# Effect of Medium Radiation on Thermal Conductivity Measurement of Aerogels Using Steady-State Heating Method

**DOI:** 10.3390/gels12060507

**Published:** 2026-06-07

**Authors:** Fengfei Lou, Sujun Dong, Xia Liu, Haitao Fan, Xun Wang, Keyong Zhu, Yinwei Ma

**Affiliations:** 1 School of Aeronautics, Shanghai DianJi University, Shanghai 201306, China; lloufengfei@163.com (F.L.); 32121@sdju.edu.cn (H.F.); wangxunweihua@163.com (X.W.); 2 School of Aeronautic Science and Engineering, Beihang University, Beijing 100191, China; dsj@buaa.edu.cn; 3 Research Institute of Semiconductor Equipment, Beijing 100176, China; 15210857193@163.com; 4 Key Laboratory of Modern Acoustics, Ministry of Education, Nanjing University, Nanjing 210093, China; 5 Department of Energy and Power Engineering, Tsinghua University, Beijing 100084, China; 6 National Key Laboratory of Aerospace Flight Technology, Beijing 100074, China

**Keywords:** semi-transparent materials, silica aerogel composite, steady-state heating method, effective thermal conductivity, thermal conductivity measurement, conduction-radiation coupling, medium radiation, coupling interface emissivity, measurement error

## Abstract

Radiative heat transfer in aerogels (semi-transparent materials) acts as a participating medium, causing notable errors in conventional steady-state thermal conductivity measurements. Coupled conduction–radiation heat transfer is numerically simulated to examine the influence of variations in the heating plate-specimen interface emissivity on thermal conductivity measurements, and the simulation results are experimentally validated using test systems with differing interface emissivities. The results show that the effect of interface emissivity on effective thermal conductivity is more obvious under high temperatures and low extinction coefficients. When the average temperature is 1273 K, the emissivity decreases from 1 to 0.2, and the effective thermal conductivity with extinction coefficients of 3.5 m^−1^ and 3500 m^−1^ decreases by 76.1% and 24.1%, respectively. Experimental results show that when the hot surface temperature is 873 K, the cold surface temperature differences in different test systems can reach 30 K. The experimental results have the same trend as the steady-state simulation results, which verifies the accuracy of the numerical simulations. Quantitative analysis of the steady-state heating measurement results demonstrates the effect of medium radiation in semi-transparent materials on the obtained results. The findings contribute to a more accurate characterization of silica aerogel composites and provide new insights into the influence of radiative heat transfer on thermal conductivity evaluation in semi-transparent aerogel materials, which is important for the development and application of aerogel-based thermal insulation systems.

## 1. Introduction

Thermal protection technology is an important approach to ensure the safe and reliable operation of structures under extreme thermal environments [1]. Under conditions involving strong heat flux and long-term thermal exposure, the external surface temperature of structures may rise significantly, which can cause serious damage to internal components and devices [2,3]. Therefore, thermal protection systems are considered one of the key technologies for maintaining structural integrity and operational safety in high-temperature environments [4]. In the composition of thermal protection system, aerogel composites, lightweight and efficient super thermal insulation materials, have been the subject of more and more attention in recent years [5,6]. However, the pore sizes and structural features are typically in the micro- to nanoscale, comparable to or smaller than the mean free path of the relevant energy carrier [7,8]. Moreover, the heat transfer law in the materials has an obvious micro–nano scale effect, which has a complex heat transfer mechanism different from that of general porous materials [9,10].

Aerogel composites are characterized by micro- to nanoscale heat transfer mechanisms, as well as notable semi-transparent properties [11]. Radiative heat transfer within the material behaves as a participating medium and contributes significantly to effective thermal conductivity (ETC) at high temperatures [12,13]. Especially for silica aerogel, it has strong transmittance to near-infrared radiation with the wavelength of 3~8 μm at high temperatures, which leads to poor infrared radiation shielding abilities at high temperatures [14,15]. This question leads to significant error in the results of conventional thermal conductivity testing methods [16].

At present, the thermal insulation performance of aerogels is mainly tested by thermal conduction heating method (TCHM), infrared radiation heating method (IRHM), and convection heating method (CHM), among which, TCHM is the conventional thermal conductivity measurement method [17,18]. However, due to the high experimental cost and complex operation of IRHM and CHM, the two methods have limited applications and are rarely used in actual research [16]. Several typical studies will be briefly reviewed below to demonstrate the application of IRHM and CHM in measuring the thermal conductivity of semi-transparent materials.

The IRHM mainly involves heating the test piece through radiation devices such as quartz lamp, graphite, and arc lamp. Previous studies have applied this method to evaluate silica aerogels: Peng et al. [19] heated a silica composite at 1773 K with a quartz lamp, Liu et al. [20] similarly employed radiation devices to assess thermal insulation, and Ni et al. [21] used a butane flame to verify thermal protection performance.

The CHM simulates realistic operating conditions through airflow wind tunnel testing, as demonstrated by Li et al. [22], who evaluated an aerogel-based thermal protection system under combustion-gas heating, and Lou et al. [23], who showed that, although TCHM is more cost-effective, it cannot fully reproduce realistic conditions and tends to overestimate the thermal response compared to CHM.

Dai et al. [24] compared aerogels under TCHM, IRHM, and CHM, finding that IRHM produced the highest cold surface temperatures. This is due to the strong infrared transmittance of semi-transparent aerogels at high temperatures, which enhances radiative heat transfer and allows for additional energy to penetrate the material, resulting in higher measured values.

The aerogels are highly effective thermal protection materials due to excellent insulation and high-temperature resistance. The CHM can replicate realistic boundary conditions, providing measurements that closely reflect true thermal conductivity. However, the CHM is costly and operationally complex, limiting use. Most studies only record cold surface temperatures, with few performing steady-state thermal conductivity tests [23]. The limited development of high-temperature airflow testing further emphasizes the need for rigorous theoretical analysis and experimental validation of aerogel thermal conductivity.

The TCHM generally comprises two approaches, namely, the transient heating method (THM) and the steady-state heating method (SSHM) [25,26]. Owing to relatively short measurement duration and low operational cost, the THM has been widely adopted [27]. Wang et al. [28], Yang et al. [29], Cheng et al. [30], Ran et al. [31], Hu et al. [32], and Zhan et al. [33] all used this method to measure the thermal conductivity of aerogels. However, Zhang et al. [34,35,36] demonstrated that the underlying measurement principle of the THM is based on the unsteady heat conduction equation applicable to opaque media, which may introduce significant errors. This limitation becomes more pronounced at elevated temperatures and in materials with low extinction coefficients.

For semi-transparent materials such as aerogels, the SSHM (a steady-state heating method within TCHM) is currently the most accurate and cost-effective method [34,35,36], calculated using the one-dimensional Fourier heat conduction formula [23]. Lee et al. [37], Kan et al. [38], Lakatos et al. [39], and Bravo-Moncayo et al. [40] all used this method to measure the thermal conductivity of aerogels. Lou et al. [41] conducted systematic steady-state thermal conductivity tests across different temperatures and pressures. Although SSHM provides high accuracy, it cannot replicate the actual boundary conditions of aerospace applications, and the advantages and limitations of the three testing methods are summarized in Table 1.

According to GB/T 10294-2008 [42], the SSHM requires that all contact surfaces to have an emissivity of at least 0.8. Because radiative transfer within the medium significantly influences the measurement, the SSHM may encounter similar issues as IRHM, with additional heat penetrating the aerogel and causing overestimation. For researchers unfamiliar with heat transfer mechanisms of semi-transparent materials, neglecting internal radiation can lead to deviations between measured thermal conductivity and actual thermal insulation performance in aerospace applications.

To sum up, although the SSHM is widely used in thermal conductivity testing of aerogel due to high measurement accuracy and low testing cost, there is still a lack of systematic understanding of the effect of internal medium radiation of semi-transparent materials on the measurement techniques of the SSHM. Existing research mainly focuses on the characterization and comparison of the thermal protection performance of aerogels under different heating methods. However, the thermal radiation penetration effect during the SSHM and the influencing mechanism on the deviation of the measurement results have not yet been fully revealed. In particular, there are still few research reports on the influence of coupling interface emissivity on the measurement of the ETC of aerogels under different temperatures and extinction coefficients.

Silica aerogel composites are among the most widely studied aerogel-based thermal insulation materials and have attracted considerable attention for thermal protection applications in aerospace, energy, and high-temperature engineering fields. Thermal insulation performance is closely related to the coupled effects of solid conduction, gaseous conduction, and radiative heat transfer. Recent studies have demonstrated that thermal transport characteristics, radiative transfer behavior, and structure–property relationships play critical roles in determining the thermal performance of aerogels [43,44,45,46]. Consequently, thermal conductivity is widely recognized as one of the most important parameters for evaluating aerogel insulation performance and guiding material design and practical applications. However, owing to the semi-transparent property of silica aerogels, radiative heat transfer may significantly influence the measured thermal conductivity, particularly at elevated temperatures. Therefore, understanding how radiative transfer affects thermal conductivity characterization is essential for the accurate evaluation, comparison, and application of aerogel materials. Improving the understanding and accuracy of thermal conductivity measurements is not only important for thermal performance assessment, but also represents an important aspect of aerogel characterization and application research.

In this study, a numerical model coupling heat conduction and thermal radiation is established to systematically investigate how variations in the emissivity of the coupling interface between the heating plate and aerogel specimen affect the measurements of the SSHM. The simulation results are validated experimentally using test systems with different interface emissivities. Based on the radiative transfer mechanisms within the medium, the influence of internal radiation on SSHM measurements is quantitatively analyzed, and the interplay of interface emissivity, temperature, and extinction coefficient on ETC is elucidated. This work highlights the significant role of radiative heat transfer in aerogels and provides a theoretical foundation for accurately measuring the thermal conductivity of semi-transparent materials.

## 2. Results and Discussion

### 2.1. Simulation Results of the Influence of Emissivity Change on Aerogels of Different Extinction Coefficients

**Figure 1:**
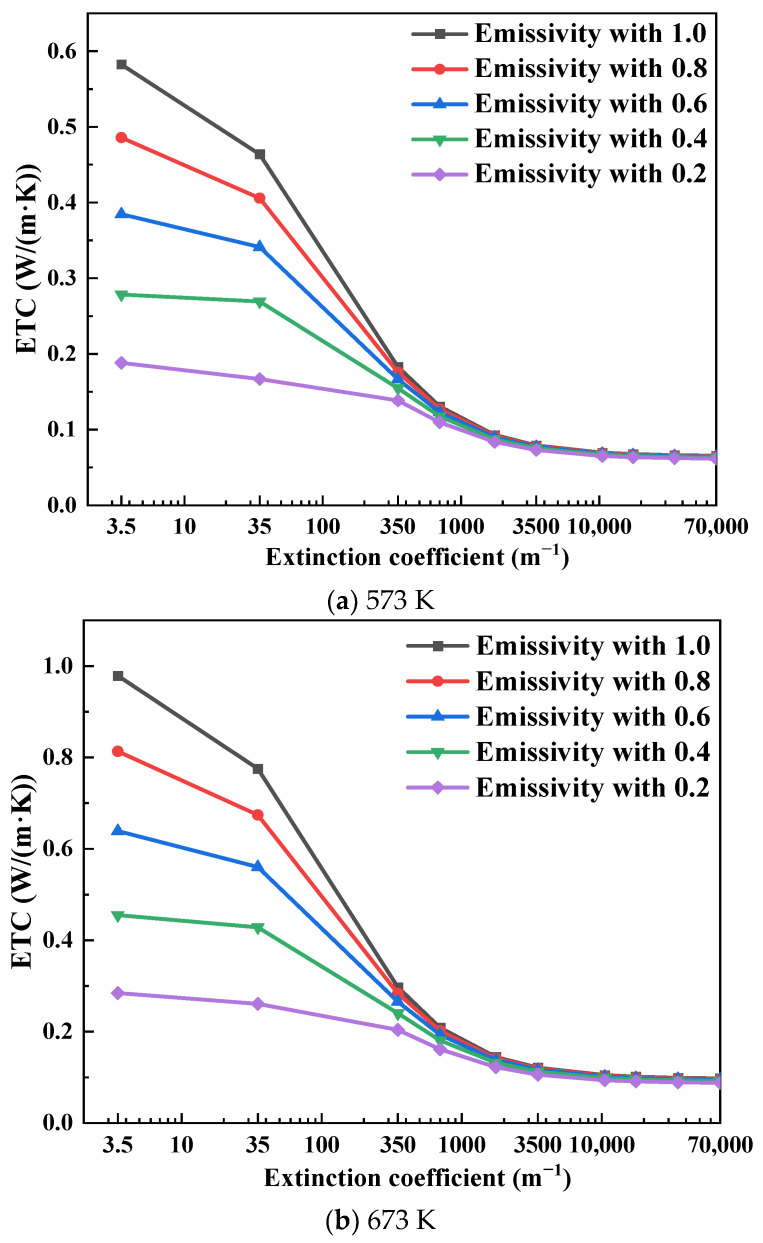

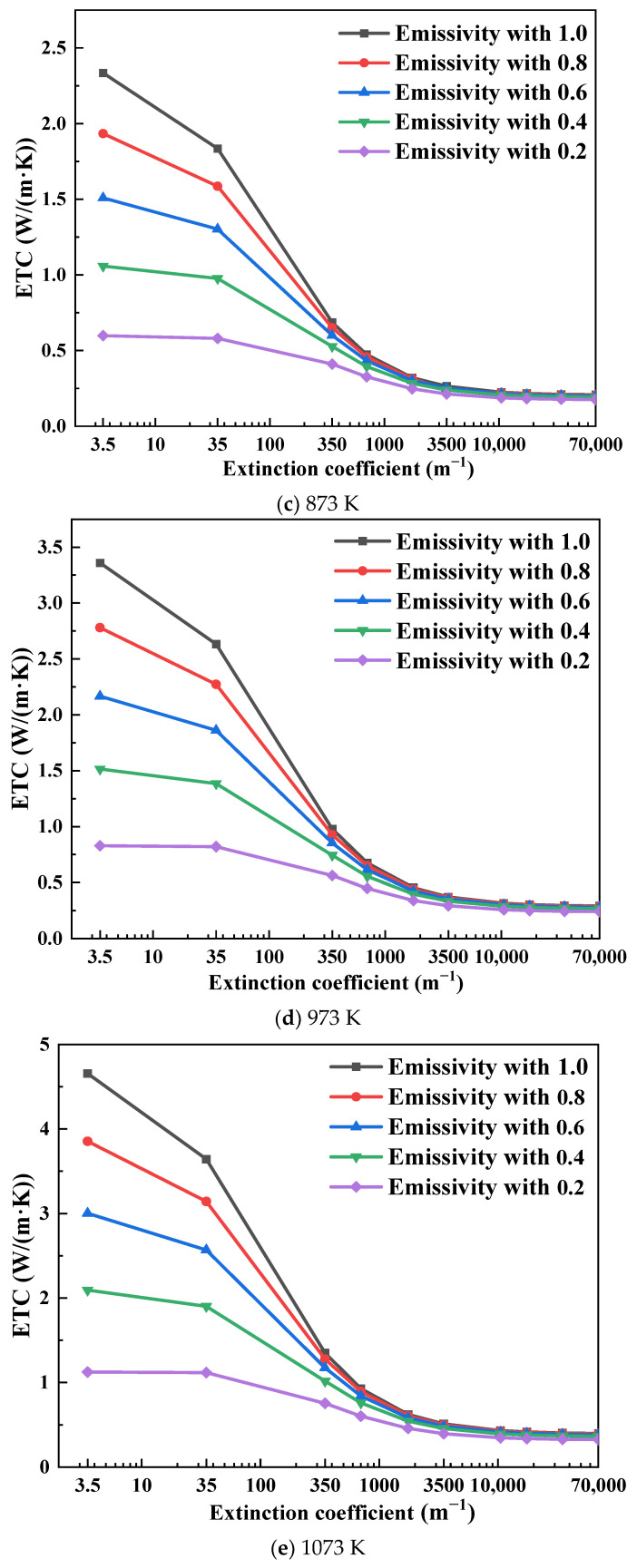

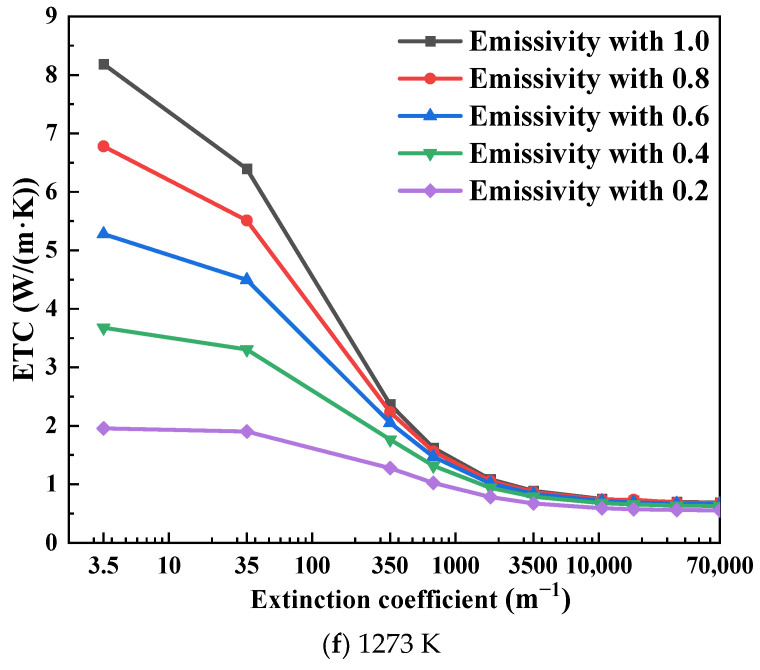
Variation in ETC with extinction coefficient at different average temperatures.

When the average temperatures are 573 K, 673 K, 873 K, 973 K, 1073 K, and 1273 K, with emissivity values of 0.2, 0.4, 0.6, 0.8, and 1, the variation in the ETC of aerogels with different extinction coefficients is presented in Figure 1.

The variation in ETC in Figure 1 is essentially governed by the competition between heat conduction and participating radiative transfer within the aerogel. For semi-transparent materials, thermal radiation is not only exchanged at the surface, but is also absorbed, emitted, and scattered inside the porous medium. Therefore, the calculated ETC is no longer determined only by solid–gas conduction, but also includes the contribution of internal radiative heat transfer. The extinction coefficient characterizes the ability of the aerogel to attenuate radiative energy. The lower extinction coefficient means stronger radiation penetration, leading to a larger radiative contribution to the total heat flux and thus a higher ETC.

As shown in Figure 1a, at an average temperature of 573 K and an interfacial emissivity of 1, the ETC values are 0.58 W/(m·K) and 0.065 W/(m·K) for extinction coefficients of 3.5 m^−1^ and 70,000 m^−1^, respectively. When the extinction coefficient increases from 3.5 m^−1^ to 70,000 m^−1^, the ETC decreases by 88.8%. At 1273 K (Figure 1f) under the same conditions, the ETC decreases by 91.6%. These results indicate that ETC decreases with increasing extinction coefficient, and the effect becomes more pronounced at higher temperatures.

This trend can be explained by the attenuation of radiative transport inside the semi-transparent medium. When the extinction coefficient is small, radiative energy can penetrate through a relatively long distance inside the aerogel, which increases the total heat flux under the same temperature difference. As the extinction coefficient increases, the absorption and scattering effects are progressively enhanced, and the radiation penetration depth is reduced. Consequently, the radiative component of heat transfer is suppressed, resulting in a lower ETC. The temperature dependence is also reasonable because radiative heat transfer increases rapidly with temperature. Therefore, the influence of extinction coefficient becomes more pronounced at higher temperatures.

As shown in Figure 1, when the extinction coefficient reaches 10,500 m^−1^ (optical thickness is 150), the ETC becomes nearly insensitive to further increases in the extinction coefficient, even at high temperatures. At an average temperature of 1273 K with an emissivity of 1, the ETC decreases by only 3.74% and 6.42% as the extinction coefficient increases to 17,500 m^−1^ and 35,000 m^−1^, respectively, relative to the case of 10,500 m^−1^.

This indicates that the aerogel gradually approaches an optically thick state when the extinction coefficient is sufficiently high. For aerogels with different extinction coefficients, the optical thickness is the product of the extinction coefficient and the characteristic thickness of the specimen. Theoretically, when the optical thickness is much greater than 1, aerogels are called optically thick media, whereas those below 1 are called optically thin media. For optically thick media, the internal radiation is strongly attenuated within a short distance, and the radiative transfer process behaves more similarly to a diffusion-like process. Therefore, further increasing the extinction coefficient does not significantly change the total heat flux. Similar conclusions have been reported for transient hot wire and hot strip methods, where the deviation caused by radiation decreases with increasing extinction coefficient and becomes negligible for optically thick materials [34,35,36].

**Figure 2:**
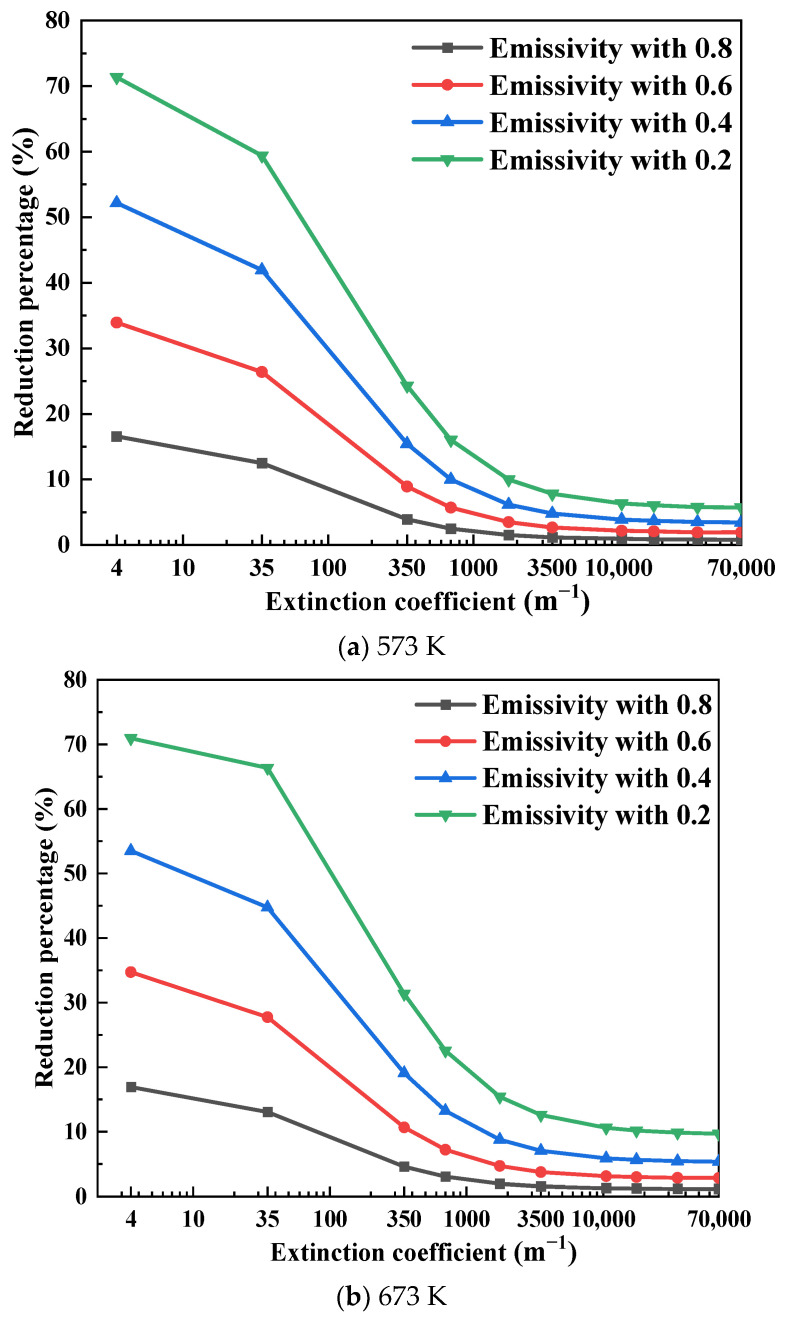

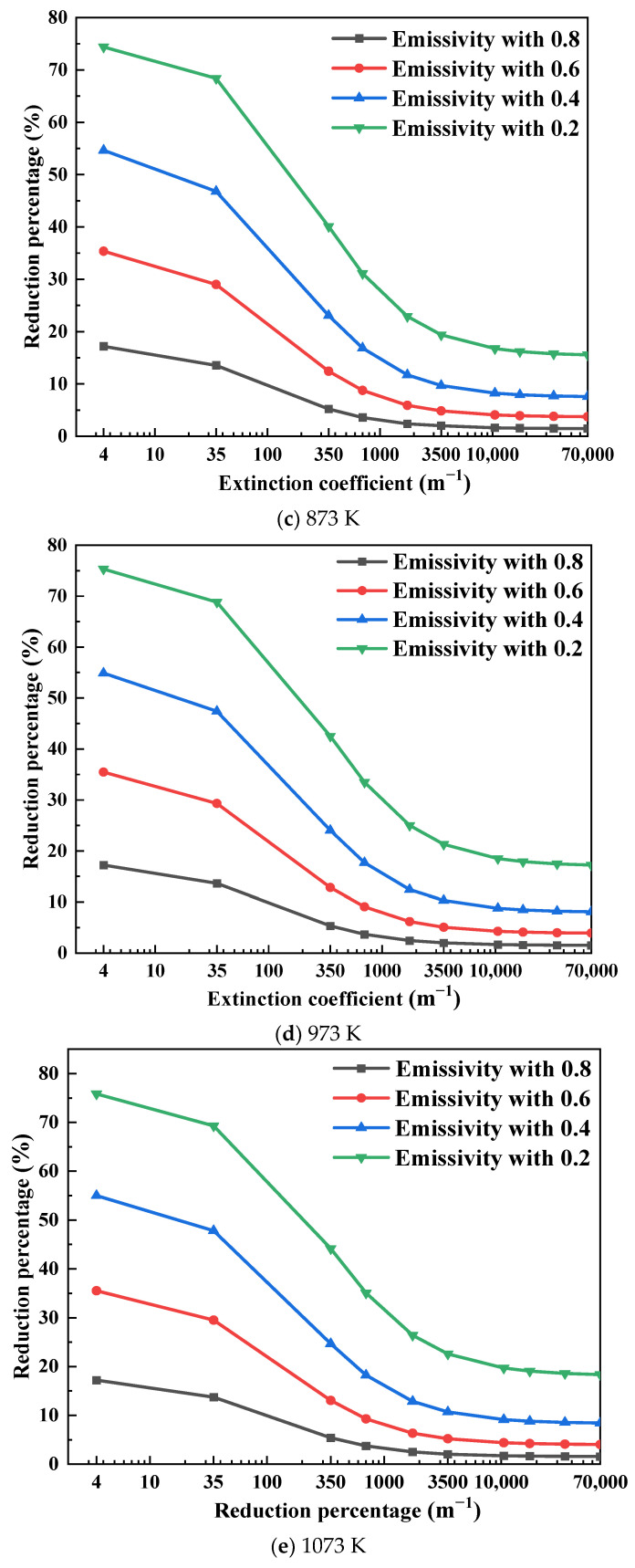

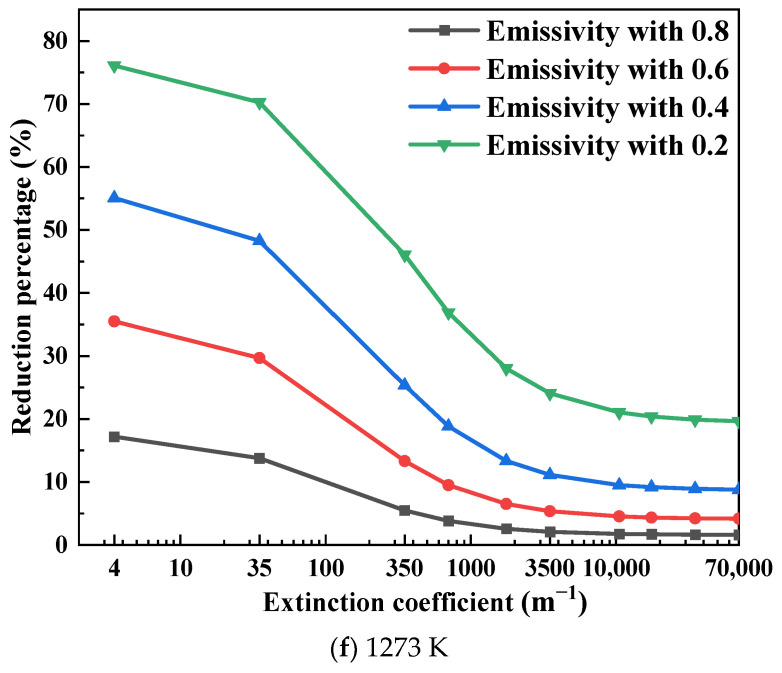
Reduction percentage of ETC relative to an emissivity of 1.

As shown in Figure 2, the influence of emissivity on ETC decreases as the extinction coefficient increases and becomes negligible when the extinction coefficient exceeds 10,500 m^−1^. For example, at 573 K (Figure 2a), reducing the emissivity from 1 to 0.8 leads to ETC reductions of 16.6%, 3.90%, 1.20%, 0.90%, 0.84%, and 0.80% for extinction coefficients of 3.5 m^−1^, 350 m^−1^, 3500 m^−1^, 10,500 m^−1^, 35,000 m^−1^, and 70,000 m^−1^, respectively.

The influence of emissivity on ETC decreases progressively as the extinction coefficient increases. The effect of coupling interface emissivity is mainly related to the radiative exchange between the heating plate and the aerogel. A higher interface emissivity enhances the emission and absorption of thermal radiation at the contact boundary, allowing for more radiative energy to enter the semi-transparent aerogel. When the aerogel has a low extinction coefficient, the radiative energy can propagate further inside the material, increasing the apparent heat flux and leading to a higher ETC. In contrast, when the extinction coefficient is large, most of the radiative energy is attenuated near the interface, so the influence of interface emissivity becomes weak.

As can be seen from Figure 2f, when the average temperature is 1273 K and the extinction coefficient is 3.5 m^−1^ and 70,000 m^−1^, respectively, the ETC of aerogels decreases by 17.2%, 35.5%, 55.1%, 76.1% and 1.62%, 4.17%, 8.79%, and 19.6%, respectively, as the emissivity decreases from 1 to 0.8, 0.6, 0.4, and 0.2. The results indicate that, under high-temperature conditions and low extinction coefficients of materials, thermal insulation performance is highly sensitive to changes in interfacial emissivity.

The above results are consistent with previous studies on transient thermal conductivity measurement of semi-transparent materials. Zhang et al. [35] reported that transient hot wire measurements may overestimate the thermal conductivity of low-extinction materials at high temperatures because the theoretical model assumes pure heat conduction, whereas the actual process involves coupled conduction–radiation heat transfer. Similar behavior was also observed for the hot strip method, where large discrepancies occurred for materials with low extinction coefficient, while the deviation decreased significantly as the extinction coefficient increased [36]. Compared with these transient-method studies, the present work further demonstrates that even in SSHM, which is generally regarded as a more accurate steady-state method, the measured ETC can still be affected by radiative boundary conditions, especially the coupling interface emissivity.

Overall, variations in coupling interface emissivity significantly affect the thermal insulation behavior of optical thin media. Because the absorption coefficient and scattering coefficient are small for optical thin media, the absorption and scattering effects of radiation inside the media are not obvious. Due to the long penetration depth of thermal radiation, radiative transfer plays a significant role in the thermal insulation behavior of materials. Especially at higher temperatures, the radiation thermal conductivity dominates the ETC of aerogels, so the decrease in emissivity is more obvious to improve the thermal insulation performance of semi-transparent materials.

However, as the extinction coefficient increases, the influence of emissivity on ETC is progressively weakened. The reason for this phenomenon is mainly because the material with a large extinction coefficient (optical thick medium) has a strong inhibitory effect on thermal radiation. Under these conditions, the coupled conduction–radiation heat transfer in an optically thick medium can be treated as a diffusion-dominated process. As a result, radiative transfer within semi-transparent materials, such as aerogels, has a negligible influence on the ETC. Therefore, compared with optical thin media, the change in coupling interface emissivity has less of an influence on optical thick media.

### 2.2. Simulation Results of Influence of Emissivity Change on ETC with Average Temperatures

**Figure 3:**
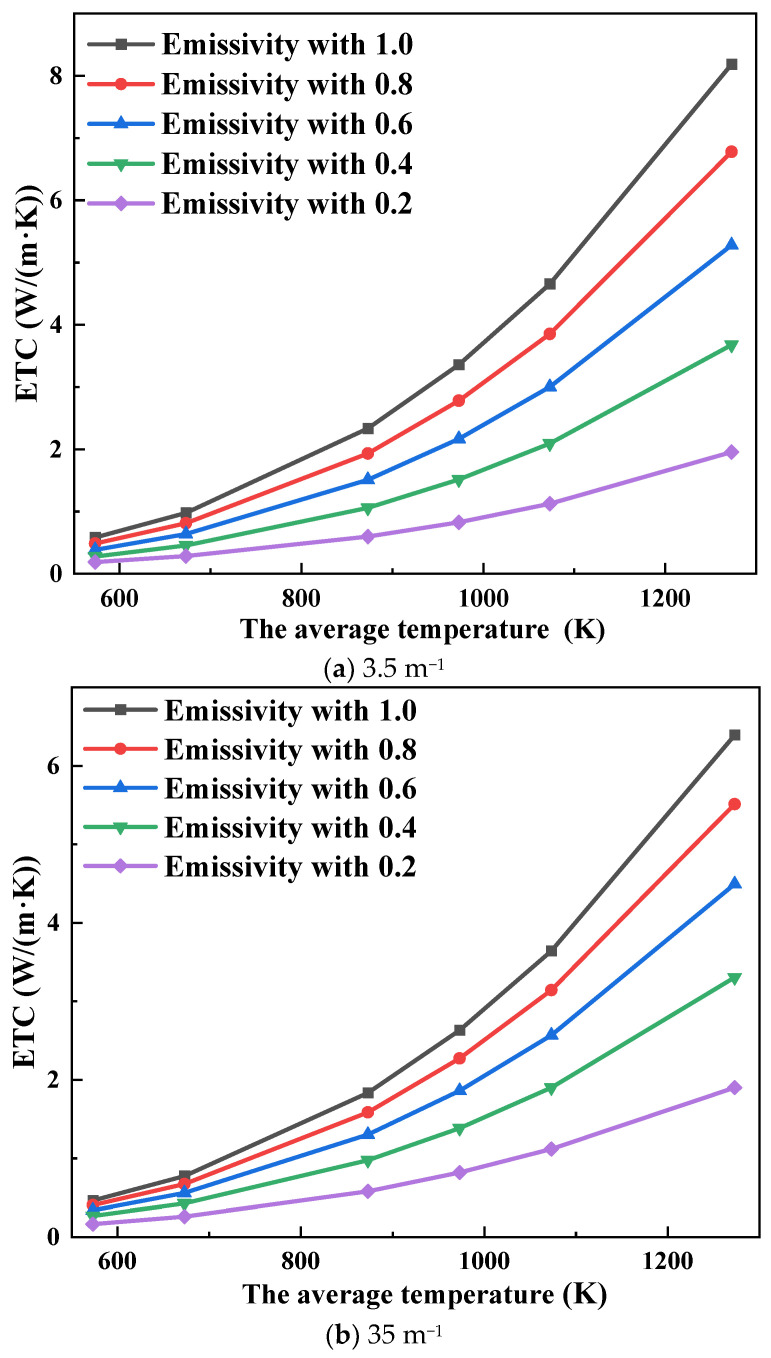

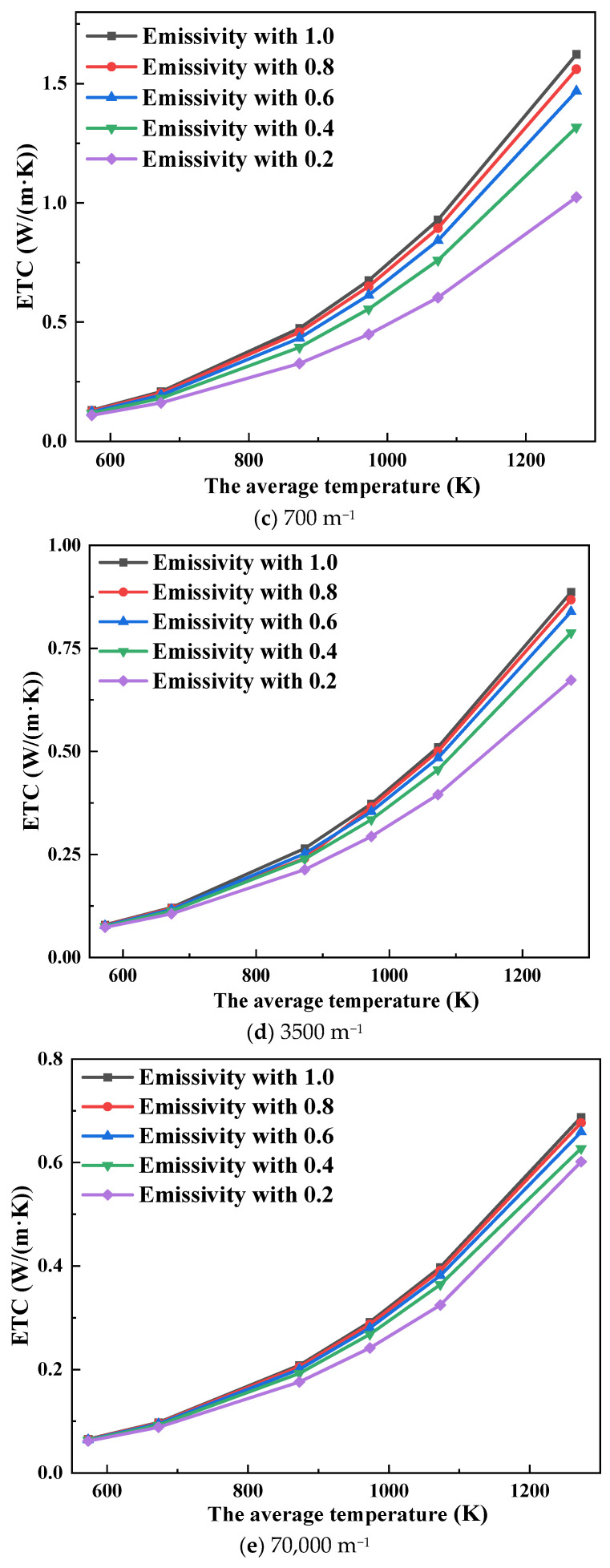
Variation in ETC with average temperature.

As shown in Figure 3, the ETC of aerogels varies with the average temperature when the extinction coefficients are 3.5 m^−1^, 35 m^−1^, 700 m^−1^, 3500 m^−1^, and 70,000 m^−1^ at different coupling interface emissivities. As can be seen from Figure 3, the ETC of aerogels increases with an increase in temperature, and the rate gradually increases.

Under different extinction coefficients, with the increase in temperature, the influence of the decrease in coupling interface emissivity on the ETC increases gradually. At the extinction coefficient of 35 m^−1^ and average temperatures of 573 K, 673 K, 1073 K, and 1273 K, the ETC decreases by 64.1%, 66.3%, 69.3%, and 70.3%, respectively, as the emissivity is reduced from 1 to 0.2. The main reason for this phenomenon is that the higher the temperature, the greater the radiation heat transfer inside the material, so the decrease in emissivity is more obvious to improve the thermal insulation performance of aerogels.

However, as can be seen in Figure 3e, when the extinction coefficient is large, even if the temperature is high, the change in the coupling interface emissivity has a minor influence on the ETC. At an extinction coefficient of 70,000 m^−1^ and an average temperature of 1273 K, the ETC decreases by only 1.60% as the emissivity is reduced from 1 to 0.8. This phenomenon is mainly due to the optical thickness of the aerogels with large extinction coefficients being large, and the radiation heat in the medium only penetrates a short distance, so the influence of radiation heat transfer inside the material is small. Therefore, even if the temperature is high, the change in emissivity has a minor effect on the ETC of aerogels.

As can be seen in Figure 3d, when the extinction coefficient of an aerogel is 3500 m^−1^ (optical thickness is 52), the average temperatures are 573 K and 1273 K, respectively, and the emissivity is reduced to 0.8, 0.6, 0.4, and 0.2, respectively, and the ETC decreases by 1.16%, 2.68%, 4.79%, and 7.80% and 2.09%, 5.35%, 11.1%, and 24.1%, respectively, as the emissivity is reduced from 1 to 0.2.

For aerogels with an optical thickness of 52, the ETC is highly sensitive to changes in coupling interface emissivity. Especially at high temperatures, the influence on thermal insulation performance improvement is more obvious. Because the SSHM requires that the emissivity of the contact surface between the heating surface and the test specimen must be above 0.8, even if a material with a large optical thickness is measured by the SSHM, the influence of the medium radiation of the material on the measurement results cannot be ignored.

To sum up, because the test environment of SSHM is completely different from the real boundary conditions when aerogel is used as thermal protection material, the thermal conductivity measured by this method is different from the thermal insulation performance of aerogel in actual operation (aerospace field). In order to solve this question, the numerical simulation calculation of heat conduction and thermal radiation coupling heat transfer in the testing process of SSHM was carried out. Through the quantitative analysis of the simulation results, it can be pointed out that this method is not suitable for measuring the thermal conductivity of thermal protection materials such as aerogels. The research content of this paper reveals the influence of medium radiation in semi-transparent materials on the measurement results of SSHM, which provides theoretical guidance for the accurate measurement of thermal conductivity of thermal protection materials.

### 2.3. Experimental Results of Temperature Rise Curve of Cold Surface

**Figure 4:**
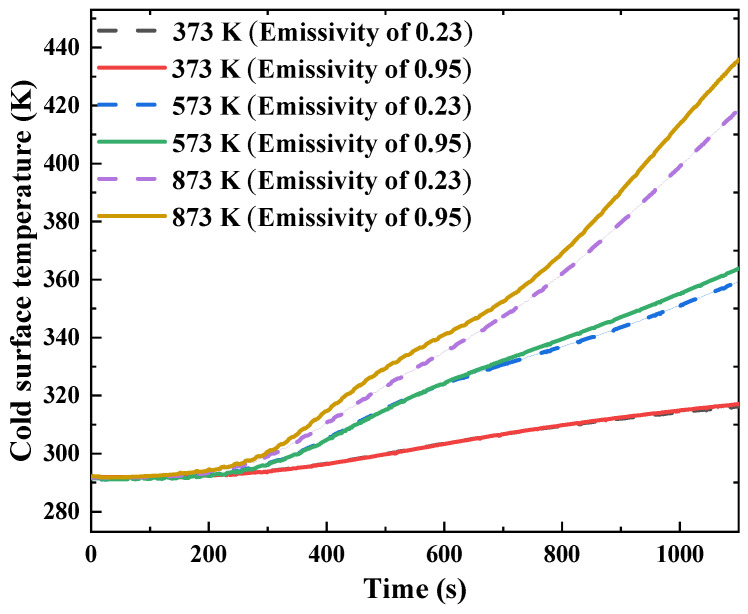
Cold surface temperature rise curve of aerogel.

When the hot surface temperatures are 373 K, 573 K, and 873 K, respectively, the cold surface temperature rise curve of aerogel is as shown in Figure 4. At the hot surface temperature of 373 K, only negligible differences are observed in the cold surface temperature rise curves for emissivities of 0.23 and 0.95. However, with the increase in temperature, the difference in the temperature-rise curve of the cold surface gradually increases.

Compared to the test system with a hot surface temperature of 373 K, when the hot surface temperature is 573 K, the cold surface temperature-rise curves of aerogels under the two test systems show a minor difference. However, when the hot surface temperature is 873 K, the difference in the temperature-rise curve of the cold surface is more obvious. Under this heating condition, when the test runs to 1100 s, the temperature difference between the two testing systems can reach 30 K. The main reason for this phenomenon is that the higher the temperature, the greater the influence of the medium radiation of aerogels on the overall thermal insulation performance. Therefore, with the increase in temperature, the decrease in the emissivity is more significant for improving the overall thermal insulation performance of the material, and the temperature difference between the two test systems gradually increases with the increase in temperature. The experimental results show the same trend as the steady-state simulation results, which proves the accuracy of numerical simulation.

More importantly, the experiment confirms the mechanism revealed by the simulation. At 373 K, radiative heat transfer is relatively weak, so changing the interface emissivity has a minor influence on the cold-side temperature. As the hot-side temperature increases to 873 K, the radiative contribution becomes stronger, and the high-emissivity interface allows for more radiative energy to be transferred into and through the aerogel. Therefore, the cold-side temperature of the high-emissivity system becomes significantly higher. Based on the above analysis, the deviation in SSHM is mainly caused by internal participating radiation rather than by ordinary conductive heat transfer.

Overall, both the numerical and experimental results indicate that the ETC measured by SSHM is not only a material-related parameter, but also a boundary-condition-dependent apparent value for semi-transparent aerogels. Under high-temperature conditions, the internal radiation contribution is enhanced and the coupling interface emissivity directly affects the amount of radiative energy entering the material. Therefore, when SSHM is used for aerogel thermal conductivity measurement, the radiative properties of both the material and the contact interface should be carefully considered. This also explains why different heating methods may give different ETC values for aerogels. Previous CHM and TCHM comparisons showed that the ETC measured by CHM is lower than that measured by TCHM under comparable average temperatures [23]. This difference is consistent with the present mechanism: different heating boundaries introduce different levels of radiative participation, leading to method-dependent ETC values.

## 3. Conclusions

In this paper, the influence of medium radiation on the thermal conductivity measurement of aerogels using SSHM is systematically investigated using numerical simulation and experimental validation. The major results can be summarized as follows:

(1) The ETC obtained by SSHM is significantly affected by the extinction coefficient of aerogels. When the extinction coefficient increases from 3.5 m^−1^ to 70,000 m^−1^, the ETC decreases markedly, indicating that internal radiative heat transfer plays a dominant role for low-extinction materials. Moreover, this effect becomes more pronounced at elevated temperatures due to the strong temperature dependence of radiation.
(2) The coupling interface emissivity between the heating plate and the aerogel has a substantial impact on the measurement of SSHM. At high temperatures and low extinction coefficients, decreasing the emissivity from 1 to 0.2 leads to a significant reduction in ETC, demonstrating that radiative boundary conditions directly influence the measurement results. In contrast, for optically thick conditions, the effect of emissivity becomes negligible.
(3) The numerical results reveal that the deviation in SSHM originates from the participating radiative transfer within the semi-transparent aerogel and interaction with boundary radiation. Therefore, the measured ETC is not an intrinsic material property, but an apparent parameter dependent on both the material’s radiative properties and interface conditions.
(4) Experimental results show consistent trends with the numerical simulations. At elevated temperatures, the cold-side temperature difference between test systems with different interface emissivity can reach up to 30 K, which confirms the reliability of the numerical model and validates the proposed mechanism.

This study demonstrates that the traditional SSHM may overestimate the thermal conductivity of semi-transparent aerogels under high-temperature conditions, especially for materials with low extinction coefficients. The findings provide important guidance for improving the accuracy of thermal conductivity measurements and for the proper interpretation of experimental data for aerogel-based thermal protection materials.

Future work will focus on developing improved measurement methods and correction models that explicitly account for coupled conduction–radiation effects. In addition, extending the present study to high-temperature convective–radiative coupled environments will be essential for establishing more accurate and application-oriented thermal property evaluation methods for aerogel composites.

## 4. Materials and Methods

### 4.1. Test Specimen–Silica Aerogel Composite

The material used is a mullite-fiber-reinforced silica aerogel composite prepared in this work. The composite consists of a SiO_2_ aerogel matrix and mullite fiber felt reinforcement. The mullite fiber felt is a synthetic ceramic fiber primarily composed of Al_2_O_3_ and SiO_2_ rather than a natural fiber.

Mullite fiber felts are widely used as reinforcement materials in high-temperature thermal insulation composites because of their excellent thermal stability, low thermal conductivity, and oxidation resistance [47,48]. The continuous service temperature of the mullite fiber felt used in this study is approximately 1473 K, which exceeds the maximum temperature investigated in the present work.

**Figure 5:**
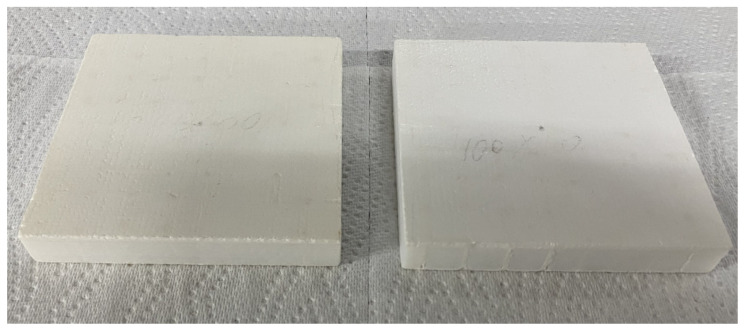
Silica aerogel composite.

The optical thickness of the silica aerogel composite used in this work is 52 (the optical thickness is the product of the characteristic thickness of material and the extinction coefficient), as shown in Figure 5, with a side length of 100 mm and a thickness of 15 mm. The surface flatness of silica aerogel composites is good, which meets the test requirements.

The SiO_2_ aerogel composite investigated in this study is described from two complementary perspectives. The synthesis route is first outlined to clarify the fabrication procedure. Subsequently, material characterization is conducted to confirm the formation of the micro–nano porous network and to ensure suitability for thermal conductivity measurements. Specifically, Scanning Electron Microscopy (SEM) is employed to examine the microstructural features, while the skeleton density and apparent density are determined to quantitatively evaluate the material properties. Additionally, N_2_ adsorption–desorption isotherms are measured to characterize the pore structure and specific surface area of the aerogel, providing further insight into its micro–mesoporous network.

(1) Preparation process

**Figure 6:**
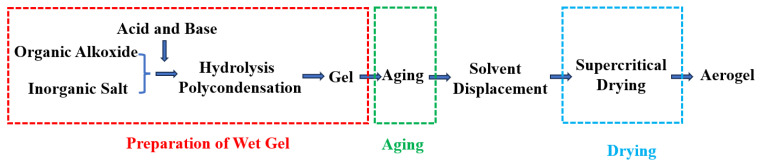
The preparation process of the SiO_2_ aerogel composite.

The fabrication route of the SiO_2_ aerogel composite is illustrated in Figure 6 and involves three main stages: gel formation, aging, and drying. The process begins with the preparation of a silica sol precursor by mixing silica sol, water, and the catalyst at a prescribed ratio to form a homogeneous gel. The precursor is then infiltrated into a mullite fiber felt (100 mm × 100 mm × 15 mm), which serves as the reinforcement phase of the composite. The mullite fiber felt is a synthetic ceramic fiber primarily composed of Al_2_O_3_ and SiO_2_ and possesses a continuous service temperature of approximately 1473 K. Subsequently, the composite undergoes aging, solvent exchange, and supercritical drying, followed by hydrophobic treatment to obtain the final mullite-fiber-reinforced SiO_2_ aerogel composite.

(2) Measurement of density and porosity

To further characterize the structural features of the investigated mullite-fiber-reinforced SiO_2_ aerogel composite, the apparent density and skeletal density are measured. The bulk volume of the specimen was determined using the Archimedes drainage method, while the mass was measured using an electronic balance. The apparent density is subsequently calculated according to Equation (1). The skeletal density is measured using an AccuPyc II 1340 automatic gas pycnometer (Micromeritics Instrument Corporation, Norcross, GA, USA), and the porosity is calculated based on the apparent and skeletal densities according to Equation (2) [49].

The measured apparent density and skeletal density are 373 kg/m^3^ and 2466.4 kg/m^3^, respectively, corresponding to a porosity of 84.9%. These parameters provide important structural information for the investigated aerogel composite and facilitate the reproducibility and comparison of thermal conductivity measurements. Although silica aerogels typically exhibit porosities approaching 90% [50], the porosity obtained in the present study is slightly lower. This difference can be attributed to the incorporation of mullite fibers, which enhance the structural integrity and high-temperature stability of the composite while partially reducing the overall pore volume.

(1) $$\rho_{\mathrm{por}}=\frac{m}{V}$$

(2) $$\phi =(1-\frac{\rho_{\mathrm{por}}}{\rho_{s}})\times 100\%$$

where *m* is the mass, *V* is the volume, $\rho_{\mathrm{por}}$ is the apparent density, $\rho_{s}$ is the skeleton density, and ϕ is the porosity.

(3) Performance characterization—SEM

**Figure 7:**
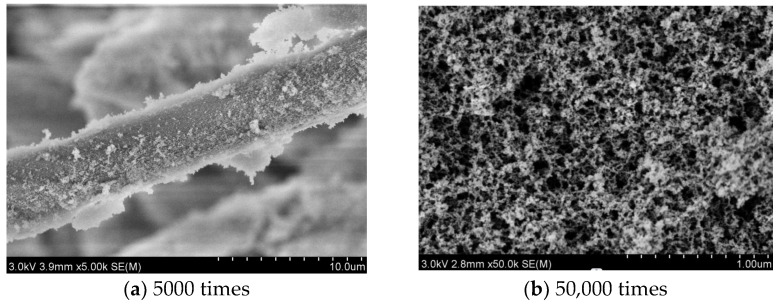
Microstructure of SiO_2_ aerogel composite.

SEM is employed to examine the microstructural features of the SiO_2_ aerogel composite using a SU8020 instrument (Hitachi High-Tech Corporation, Tokyo, Japan). The obtained images are presented in Figure 7.

At low magnification (Figure 7a), the reinforcing mullite fibers with an average diameter of approximately 5 μm are uniformly embedded within the silica aerogel matrix. The observed structure confirms the successful fabrication of the mullite-fiber-reinforced silica aerogel composite investigated in this study. At higher magnifications (Figure 7b), aggregated particles form nanoporous structures, which suppress gas-phase heat transfer. The intact fiber–particle network also indicates good structural stability at high temperatures.

(4) N_2_ Adsorption–Desorption Isothermal Curve

**Figure 8:**
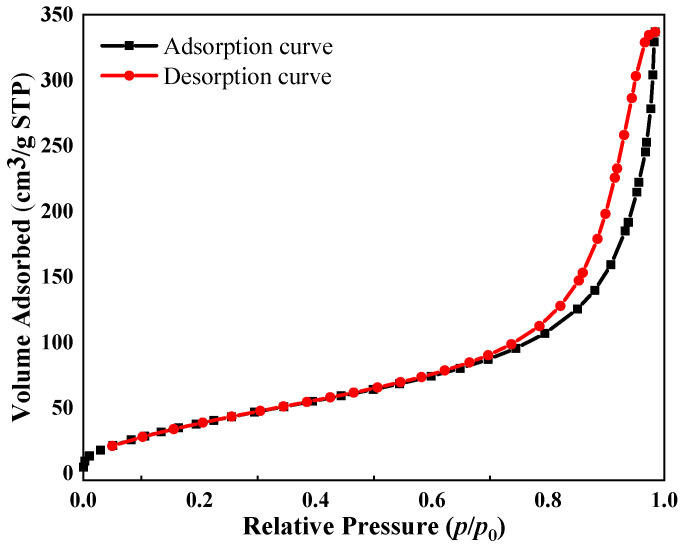
N_2_ adsorption–desorption isothermal curve.

**Figure 9:**
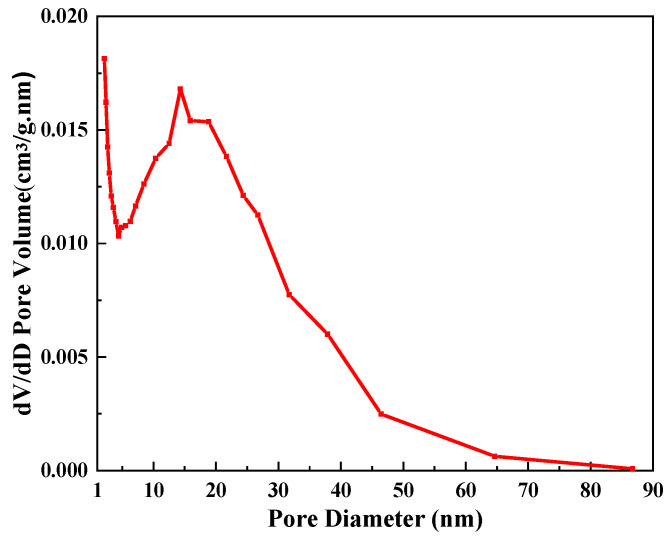
Pore size distribution curve.

The pore structure of the SiO_2_ aerogel composite was examined using an APAS-2460 physical adsorption analyzer (American Mack Company, Norcross, GA, USA). The sample was degassed at 473 K for 6 h prior to N_2_ adsorption–desorption measurements. The specific surface area was determined by the BET method, and pore size distribution was analyzed using the BJH method. The results (Figure 8) indicate a type IV isotherm according to IUPAC classification, characteristic of mesoporous materials, with a pore size distribution centered around 16 nm and a BET surface area of 568.7 m^2^/g (Figure 9).

(5) Material composition and basic characteristics of the silica aerogel composite

Based on the above characterization results, the material composition and key structural characteristics of the investigated silica aerogel composite can be clearly identified. For clarity, the relevant information is summarized in Table 2.

The results summarized in Table 2 indicate that the investigated material is a typical mullite-fiber-reinforced silica aerogel composite with a highly porous micro–nano structure, making it representative of the aerogel-based thermal insulation materials used in high-temperature environments.

### 4.2. Numerical Modeling

#### 4.2.1. Numerical Simulation Method

##### Overall Method

(1) Measurement Principle of SSHM

Within the framework of SSHM, heat transfer is predominantly governed by heat conduction, resulting in a time-invariant temperature field in the sample. Considering the one-dimensional characteristics of heat transfer, the temperature distribution can be simplified and formulated as Equation (3). The heat in the test sample is transferred along the temperature gradient direction, and the thermal conductivity of the test sample can be calculated according to the one-dimensional Fourier heat conduction law, as shown in Equation (4).

(3) T=f(y)

(4) $$\lambda_{\mathrm{eff}}=\frac{q\delta }{\Delta T}$$

where *T* is the temperature, $\lambda_{\mathrm{eff}}$ is the ETC, *q* is the heat flux density, δ is the characteristic thickness, and ΔT is the temperature difference between the cold surface and the hot surface of the test sample.

(2) Conduction–Radiation Coupling Heat Transfer in Semi-Transparent Aerogels

Semi-transparent materials, including aerogels, are characterized by radiative heat transfer involving the participating medium., which is affected by extinction coefficient (extinction coefficient is the sum of absorption coefficient and scattering coefficient, which is an index to characterize the ability of aerogel to suppress radiation heat and evaluate the overall absorption and scattering effect), so the real thermal conductivity of semi-transparent materials cannot be readily determined using a simple analytical formulation. Based on this, the one-dimensional Fourier heat conduction equation is applied to determine the thermal conductivity of aerogel, defined as ETC.

It should be noted that the assumption of purely conductive heat transfer in SSHM is strictly valid for opaque materials. For semi-transparent media such as aerogels, radiative transfer within the material may contribute to the overall heat flux, particularly at elevated temperatures and low extinction coefficients.

Consequently, although the ETC is evaluated using the Fourier law within the SSHM, the actual heat transfer process during measurement involves both conduction and radiation. In this work, a coupled conduction–radiation model is adopted to describe the actual heat transfer mechanism and quantify the influence of radiative effects on the evaluated ETC.

The ETC of aerogels is calculated by one-dimensional heat transfer Fourier formula, and the main reasons are summarized as follows: firstly, the ETC obtained by the one-dimensional steady-state method has high accuracy; secondly, semi-transparent materials are usually used as thermal insulation materials in steady state; thirdly, the discrete ordinate method is suitable for the medium participation question of radiation heat transfer in both optical thick medium and optical thin medium.

In this section, a one-dimensional steady-state numerical model of coupled heat conduction and radiation is employed to investigate the effect of emissivity variations on the thermal insulation performance of aerogels. On the premise of determining the absorption coefficient, scattering coefficient, hot surface temperature (boundary temperature), and cold surface temperature (boundary temperature), the periphery of the calculation domain is set as a adiabatic boundary. Through the numerical simulation of one-dimensional steady-state coupled heat transfer of calculation models with different grid numbers, the total heat flux density through the aerogels is calculated, and the ETC is calculated by Equation (4).

##### Computational Model

The influence of interfacial emissivity variations on the thermal insulation behavior of semi-transparent absorbing-scattering media is examined in this section.

(1) Computational domain and boundary conditions

**Figure 10:**
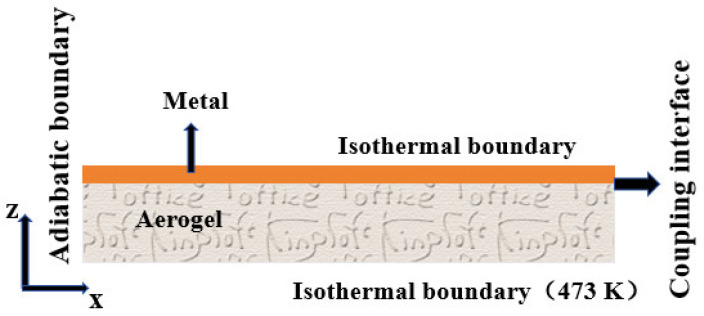
Schematic diagram of calculation domain of SSHM.

As illustrated in Figure 10, the computational domain of the aerogel is defined as a three-dimensional heat transfer model with a side length of 100 mm and a thickness of 15 mm. Meanwhile, the metal domain in contact with the aerogel is defined with a lateral dimension of 100 mm and a thickness of 3 mm. The side boundaries of the aerogel and metal domains are assumed to be adiabatic, while the isothermal boundary conditions are applied at the hot and cold surfaces, with the cold-side temperature is fixed at 473 K.

In the numerical simulation calculation, the heating plate is made of metal. The interface between the metal and aerogel is called the coupling interface, where the emissivity is called the coupling interface emissivity. Emissivity is a parameter that indicates the thermal radiation emission and absorption ability of the material. As emissivity increases, the ability of aerogels to emit thermal radiation becomes more significant. Considering the contribution of internal radiative heat transfer in the aerogel, it can be inferred that higher emissivity at the coupling interface leads to an increase in ETC.

**Figure 11:**
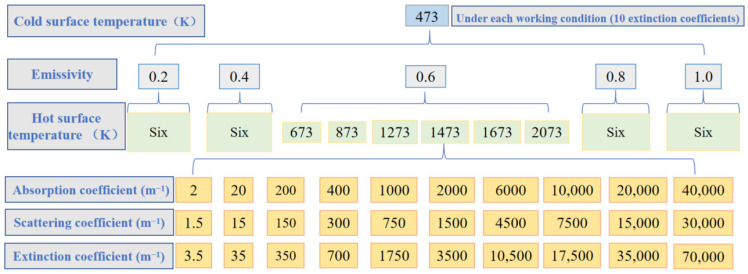
Calculation conditions of SSHM.

The calculation conditions of SSHM are shown in Figure 11. The cold surface temperature is set to 473 K, and the coupling interface emissivity is set to 0.2, 0.4, 0.6, 0.8, and 1, respectively. Under each emissivity condition, the hot surface temperatures are 673 K, 873 K, 1273 K, 1473 K, 1673 K, and 2073 K, respectively. At each hot surface temperature, aerogels are set with ten different absorption coefficients and scattering coefficients, respectively. By calculating the above working conditions, the influence of the medium radiation of the aerogel on the measurement results of SSHM is studied.

The thermophysical properties of the metal and aerogel are presented in Table 3. The refractive index of the aerogel computational domain is set to 1, and the internal emissivity of all boundaries is set to 0.86. Because the metal domain is treated as non-participating in radiative transfer, the refractive index and emissivity are not defined.

To clarify the role of the metal heating plate in the numerical model, it should be emphasized that the metal layer is primarily introduced to ensure a uniform temperature distribution at the heating boundary, rather than participate in the coupled heat transfer process within the aerogel.

Specifically, the metal plate is designed with a relatively small thickness (3 mm) and a high thermal conductivity (387.6 W·m^−1^·K^−1^), resulting in a negligible temperature gradient across the metal domain. Under such conditions, the metal layer behaves effectively as an isothermal boundary, which ensures stable and uniform thermal loading on the aerogel specimen.

Therefore, the thermophysical properties of the metal have a limited influence on the overall heat transfer behavior of the system. Variations in metal properties may slightly affect the local temperature distribution near the interface, but they do not alter the dominant heat transfer mechanisms within the aerogel, particularly the conduction–radiation coupling governed by the extinction coefficient.

As a result, the observed relationship between the ETC and extinction coefficient is determined primarily by the radiative properties of the aerogel. Changing the metal properties would not reverse or fundamentally modify the overall trend (i.e., the decrease in ETC with increasing extinction coefficient), but may only introduce minor quantitative differences in the calculated values.

(2) Verification of grid independence

The density, specific heat capacity, and thermal conductivity of the metal and aerogel are set according to Table 3. The refractive index of the aerogel is set to 1, the internal emissivity of all boundaries is set to 0.86, and the absorption coefficient and scattering coefficient are set to 2000 m^−1^ and 1500 m^−1^, respectively. The boundaries of the metal and aerogel computational domains are all set to adiabatic conditions, with the emissivity set to 1. The hot and cold boundaries are specified as isothermal surfaces with temperatures of 673 K and 473 K, respectively. Under the specified conditions, a numerical simulation of coupled heat transfer involving conduction and thermal radiation is performed, and the ETC of the aerogel is determined using Equation (4).

**Figure 12:**
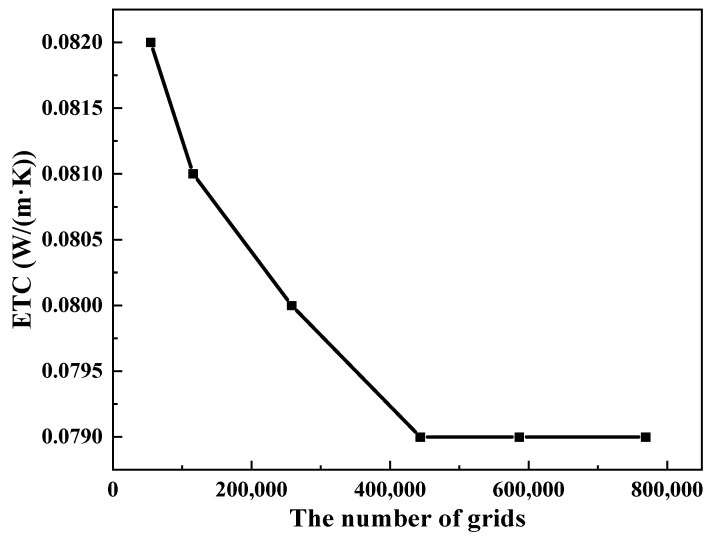
Sensitivity of the ETC to the number of computational grids.

The calculation results of ETC of aerogels with different grid numbers are shown in Figure 12. It can be seen from Figure 12 that when the number of grids is more than 443,687, the ETC calculated by using the one-dimensional heat conduction Fourier formula will no longer change with the number of grids. Therefore, the grid number of 443,687 is selected for numerical simulation calculation of coupling heat transfer of heat conduction and thermal radiation.

##### Governing Equations and Numerical Methods

(1) Governing equations

For the metal computational domain, the energy equation within the material, as shown in Equation (5), is solved using the finite volume method. Within the computational domain of semi-transparent media, the energy conservation equation is given in Equation (6). The radiative heat flux term in this equation is intrinsically linked to the radiation intensity inside the medium, as shown in Equations (7)–(10).

(5) $$\rho c\frac{\partial T(x,y,z)}{\partial t}=-\nabla \cdot q_{t}=-\nabla \cdot q_{c}=\frac{\partial }{\partial x}\left(\lambda \frac{\partial T}{\partial x}\right)+\frac{\partial }{\partial y}\left(\lambda \frac{\partial T}{\partial y}\right)+\frac{\partial }{\partial z}\left(\lambda \frac{\partial T}{\partial z}\right)$$

(6) $$\rho c\frac{\partial T(x,y,z)}{\partial t}=-\nabla \cdot q_{t}=-\nabla \cdot q_{c}-\nabla \cdot q_{r}=\frac{\partial }{\partial x}\left(\lambda \frac{\partial T}{\partial x}\right)+\frac{\partial }{\partial y}\left(\lambda \frac{\partial T}{\partial y}\right)+\frac{\partial }{\partial z}\left(\lambda \frac{\partial T}{\partial z}\right)-\nabla \cdot q_{r}$$

(7) $$q_{r}=q_{r,x}e_{x}+q_{r,y}e_{y}+q_{r,z}e_{z}$$

(8) $$q_{r,x}=\int_{\Omega =4\pi }I\xi d\Omega$$

(9) $$q_{r,y}=\int_{\Omega =4\pi }I\eta d\Omega$$

(10) $$q_{r,z}=\int_{\Omega =4\pi }I\mu d\Omega$$

where $q_{t}$, $q_{c}$, and $q_{r}$ are the total heat flux, conduction heat flux, and radiation heat flux, respectively; ρ is density, *c* is specific heat capacity, λ is thermal conductivity, *x*, *y* and *z* represent corresponding axes, respectively, and *t* is time; $q_{r,x}$ (ξ=sinθcosφ), $q_{r,y}$ (η=sinθsinφ), and $q_{r,z}$(μ=cosθ) correspond to the radiation heat flux components in the *x*, *y*, and *z* coordinates, respectively; ξ is the direction cosine along the *x* coordinate, η is the direction cosine along the *y* coordinate, and μ is the direction cosine along the *z* coordinate; θ and φ are the zenith angle and the circle angle, respectively; Ω is the solid angle; and *I* is the radiation intensity.

Among them, the $q_{t}$, $q_{c}$, and $q_{r}$ in Equation (6) are all vectors. According to the heat transfer model in this section, vectors can be divided into components on the *x*-coordinate, *y*-coordinate, and *z*-coordinate. Equations (8)–(10) are used to express the components of radiation heat flux in *x*, *y*, and *z* coordinates, respectively, in which the radiation intensity needs to be solved.

For semi-transparent media, the internal radiation intensity is governed by the radiative transfer equation, which is formulated in Equation (11). It should be noted that the radiative heat transfer inherently depends on temperature through the blackbody emission term, indicating that the radiative contribution increases with temperature. Boundary conditions assuming opaque surfaces with diffuse emission and diffuse reflection are applied in the present model, and the mathematical expression is given in Equation (12).

(11) $$\frac{dI(s,s)}{ds}=-\beta (s)I(s,s)+\kappa (s)I_{b}(s)+\frac{\sigma_{s}(s)}{4\pi }\int_{\Omega_{i}=4\pi }I(s,s_{i})\Phi \left(s_{i},s\right)d\Omega_{i}$$

(12) $$I_{w}(s)=\varepsilon_{w}\frac{\sigma T_{w}^{4}}{\pi }+\frac{1-\varepsilon_{w}}{\pi }\int_{n_{w}\cdot s_{l}<0}I_{w}(s_{i})\left|n_{w}\cdot s_{i}\right|d\Omega_{i}$$

where $I_{b}(s)$ is the radiative intensity emitted by a black body; I(s,s) represents the radiative intensity of space position r and transmission direction s which is a vector; and $\Phi \left(s_{i},s\right)$ is a scattering phase function, which is the ratio of the scattering intensity in the **s** direction caused by incident radiation in the $s_{i}$ direction to the average scattering intensity in the 4π scattering space. Here, because radiation transfer equation is related to space and direction, I(s,s), $I(s,s_{i})$, $\Phi \left(s_{i},s\right)$ in Equation (11) are all related to direction, which are vectors. $I_{w}$ is the radiation intensity of the wall; $\varepsilon_{w}$ is the emissivity of the wall; $T_{w}$ is the temperature of the wall; and $n_{w}$ is the normal vector of the wall.

(2) Numerical methods

If it is necessary to solve the heat flux field in the medium in Equation (6), the radiation intensity field must be obtained by solving Equation (11). At the same time, the radiation intensity field in Equation (11) can only be determined if the heat flux field is known. Therefore, Equations (6) and (11) should be solved alternately until the heat flux field and radiation intensity field at each time step are consistent. Among them, the energy equation and radiation transfer equation are solved by the finite volume method and discrete ordinate method, respectively.

Using the discrete ordinates method, radiation intensity is discretized in angle and space. In the three-dimensional coordinate system (*x*, *y*, *z*), Equation (11) is then discretized along the directions ($\xi^{m}$, $\eta^{m}$, $\mu^{m}$). Because the calculation model adopts opaque boundaries with diffuse emission and diffuse reflection, the discrete radiation transfer equation and boundary conditions are shown in Equations (13) and (14), respectively. Finally, the directional discrete equations are further discretized in space using the finite difference method, enabling the evaluation of the radiation intensity field.

(13) $$\xi^{m}\frac{\partial I^{m}}{\partial x}+\eta^{m}\frac{\partial I^{m}}{\partial y}+\mu^{m}\frac{\partial I^{m}}{\partial z}=-\beta I^{m}+\kappa I_{b}(r)+\frac{\sigma_{s}}{4\pi }\left[\sum_{l=1}^{N\Omega }w^{l}I^{l}\Phi^{m,l}\right]$$

(14) $$I_{w}^{m}(s)=\varepsilon_{w}\frac{\sigma T_{w}^{4}}{\pi }+\frac{1-\varepsilon_{w}}{\pi }\sum_{n_{w}\cdot s_{l}<0}w^{l}I_{w}^{l}\left|n_{w}\cdot s_{l}\right|,\;n_{w}\cdot s_{m}>0$$

where *l* and *m* represent the *l*th and *m*th solid angles in the space direction, respectively; NΩ is the total number of discrete solid angles in 4π space direction; $w^{l}$ is the integral weight coefficient; and $\Phi^{m,l}$ is the discrete scattering phase function.

In this section, the computational fluid dynamics numerical simulation method is employed to solve the governing equations. The commercial software ANSYS ICEM CFD 2026 R1 was utilized for mesh generation, while the governing equations were solved using the commercial software ANSYS Fluent 2026 R1. In the numerical implementation, the energy equation and the radiative transfer equation were discretized by means of a second-order upwind scheme. A second-order implicit scheme was applied to discretize the unsteady term of the energy equation. The iterative procedure was deemed to be converged when the residuals associated with the energy equation and the radiative transfer equation dropped below 1.0 × 10^−8^.

#### 4.2.2. Validation of the Numerical Simulation Method

Based on the above analysis, numerical methods (the energy equation and radiative transfer equation are solved by finite volume method and discrete ordinate method, respectively) are widely used in the calculation process of the semi-transparent materials such as aerogels [24,34,35,36,51,52].

**Figure 13:**
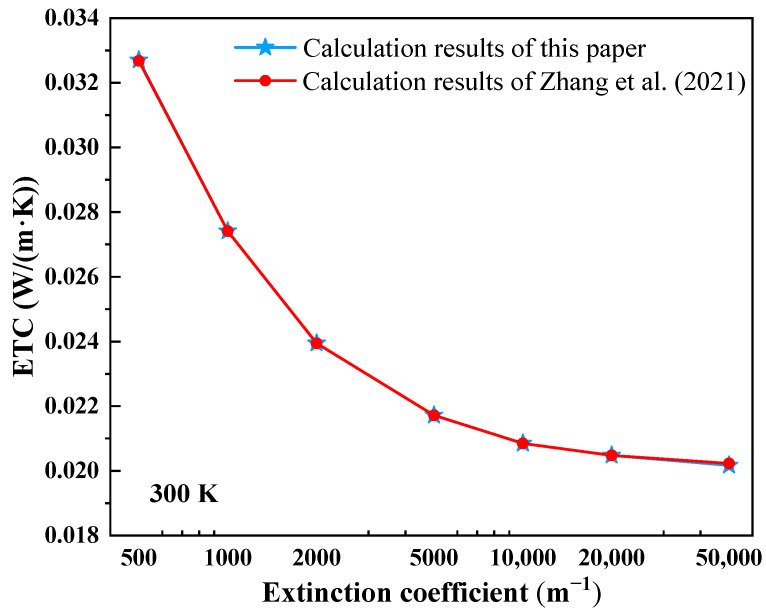
Comparison of ETC results with Zhang et al. [35].

To further assess the reliability of the present numerical approach, simulations of coupled conduction-radiation heat transfer in semi-transparent materials with different extinction coefficients are performed. The predicted results are compared with those reported by Zhang et al. [35] under identical modeling conditions, as illustrated in Figure 13. A close agreement between the two sets of results is observed, confirming the validity of the numerical method employed in this study.

### 4.3. Experimental Method

In this section, experiments are conducted using test systems with coupling interface emissivities of 0.23 and 0.95, respectively, and the influence of medium radiation on the measurement results of SSHM is analyzed. To analyze the temperature response, the aerogel is heated and the temperature at the center of the cold surface is measured, enabling a comparison of the resulting temperature rise curves.

In this work, all experiments are conducted using a mullite-fiber-reinforced silica aerogel composite. The material is characterized through SEM observation, density measurements, and N_2_ adsorption–desorption analysis to confirm microstructural features and provide essential information for the interpretation of thermal conductivity measurements.

#### 4.3.1. Emissivity Measurement of Brass Surfaces

During the test, in order to make the temperature of the specimen more uniform, a thin metal plate is placed between the ceramic heater and the specimen. The contact surface between metal and aerogel is the coupling interface, where the emissivity is the coupling interface emissivity.

**Figure 14:**
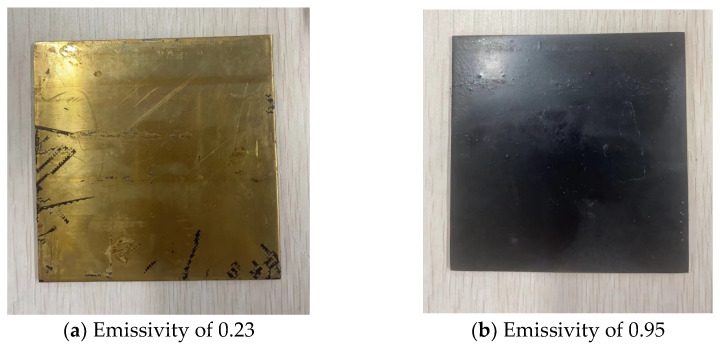
Brass samples with emissivities of 0.23 and 0.95.

During the test, the metal used is brass with an emissivity of 0.23. Brass with an emissivity of 0.95 can be obtained by spraying high-temperature-resistant black paint on brass. The brasses with emissivities of 0.23 and 0.95 are shown in Figure 14.

**Figure 15:**
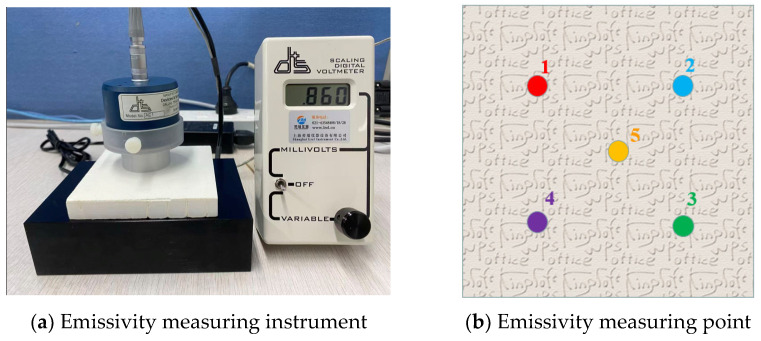
Emissivity measurement setup and measuring points.

The emissivity of aerogels was measured using the AE1/RD1 emissivity measuring instrument (Devices & Services Company, Dallas, TX, USA), as shown in Figure 15a.

**Figure 16:**
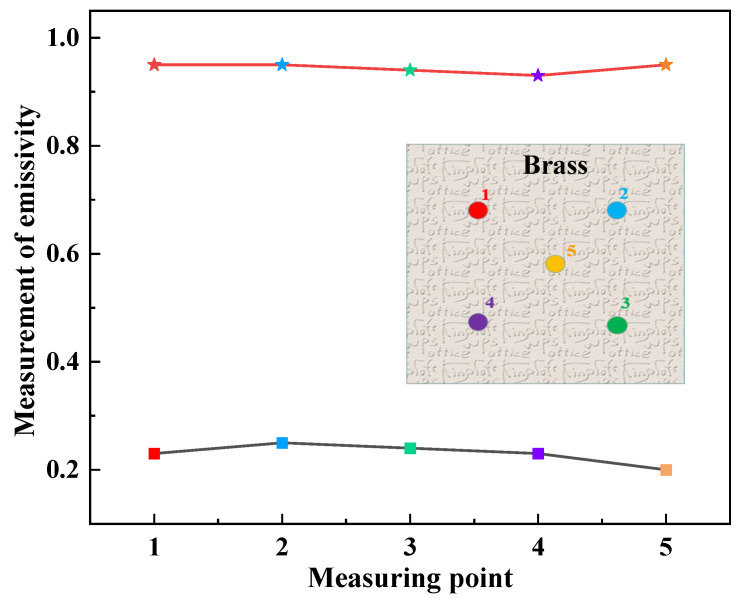
Measurement results of emissivity of two kinds of brass.

Before conducting emissivity testing, low-emissivity calibration blocks and high-emissivity calibration blocks were used to calibrate the emissivity measuring instrument. Secondly, during the test process, the detector was placed flatly on the measurement point of the sample for about 20 s, and then the detector was slid to another test point. After this cycle, the first measurement point was returned, and the final maximum value was selected as the emissivity measurement value. In order to improve the accuracy, five measuring points of the sample, labeled as points 1–5 in Figure 15b, were tested. The measured values of emissivity at five measuring points are shown in Figure 16, and the average values of emissivity at the final five measuring points of two pieces of brass are 0.23 and 0.95, respectively.

#### 4.3.2. Test Device and Method

(1) Test device

**Figure 17:**
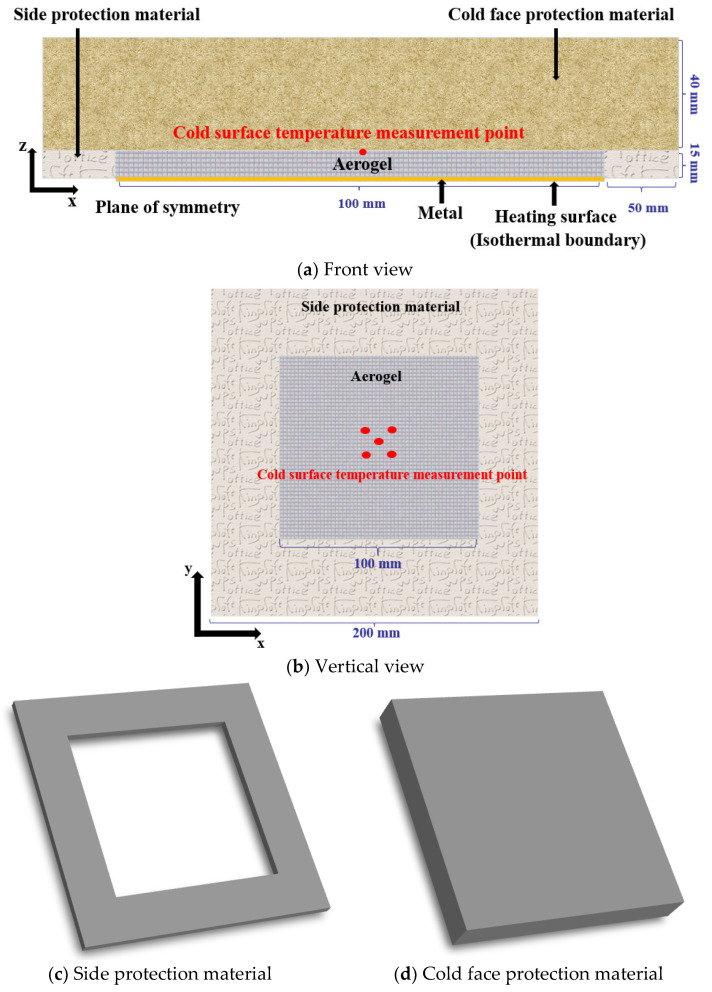
Schematic diagram of the test device.

During the test, the periphery of the aerogel is surrounded by side-protective materials, and the cold-protective materials were placed on the cold surface of the aerogel. A schematic representation of the test device is shown in Figure 17, illustrating half of the actual physical setup.

In the actual test process, the same metal brass and aerogel specimen were placed on both sides of the heating plate, and the materials with good thermal insulation performance were placed on the side and cold surface of the aerogel, respectively. In order to reduce the air thermal resistance between materials, a 10 kg ballast was placed on the test device. The heater is a ceramic heater with a side length of 100 mm. During the test, the temperature controller and power regulator were used to control the heating power of the heater so that the aerogel could be heated at different temperatures.

Because five temperature sensors are arranged on the cold surface of the aerogel, the average temperature is taken as the cold surface temperature. On this basis, first, the uncertainty of the cold surface temperature was calculated; secondly, the combined standard uncertainty was calculated, as shown in Equation (15); finally, the related expanded uncertainty was calculated (expanded factor = 2), as shown in Equation (16):

(15) $$u_{T_{\mathrm{avg}}}=\sqrt{{\left(\frac{\partial f}{\partial T_{1}}\cdot u_{T_{1}}\right)}^{2}+{\left(\frac{\partial f}{\partial T_{2}}\cdot u_{T_{2}}\right)}^{2}+{\left(\frac{\partial f}{\partial T_{3}}\cdot u_{T_{3}}\right)}^{2}+{\left(\frac{\partial f}{\partial T_{4}}\cdot u_{T_{4}}\right)}^{2}+{\left(\frac{\partial f}{\partial T_{5}}\cdot u_{T_{5}}\right)}^{2}}$$

(16) $$u_{rel,T_{\mathrm{avg}}}=\frac{u_{T_{\mathrm{avg}}}}{T_{\mathrm{avg}}}$$

where $u_{T_{\mathrm{avg}}}$ is the combined standard uncertainty and $u_{rel,T_{\mathrm{avg}}}$ is the related expanded uncertainty.

The K-type thermocouple and Agilent data acquisition instrument were used to measure and collect temperature, respectively. The details of the temperature controller (Xiamen Yudian Automation Technology Co., Ltd., Xiamen, China), power regulator (Xiamen Yudian Automation Technology Co., Ltd., Xiamen, China), K-type thermocouple, and Agilent data acquisition instrument (Agilent Technologies, Santa Clara, CA, USA) are shown in Table 4.

(2) Test method

**Figure 18:**
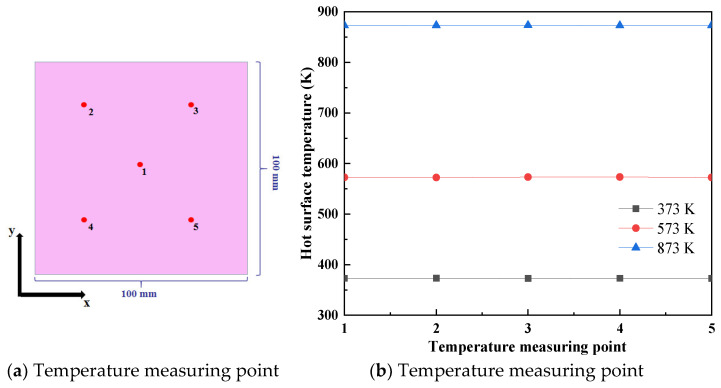
Temperature uniformity test of ceramic heating plate.

Before the test, the temperature uniformity of the ceramic heating plate was carried out. Five temperature measuring points were arranged on the hot surface of the heating plate, as shown in Figure 18. The temperature of the heating plate was set to 373 K, 573 K, and 873 K, respectively, and the temperatures of the five temperature measuring points are shown in Figure 18. It can be seen that the temperature uniformity of the heating plate is good, which meets the test requirements.

**Figure 19:**
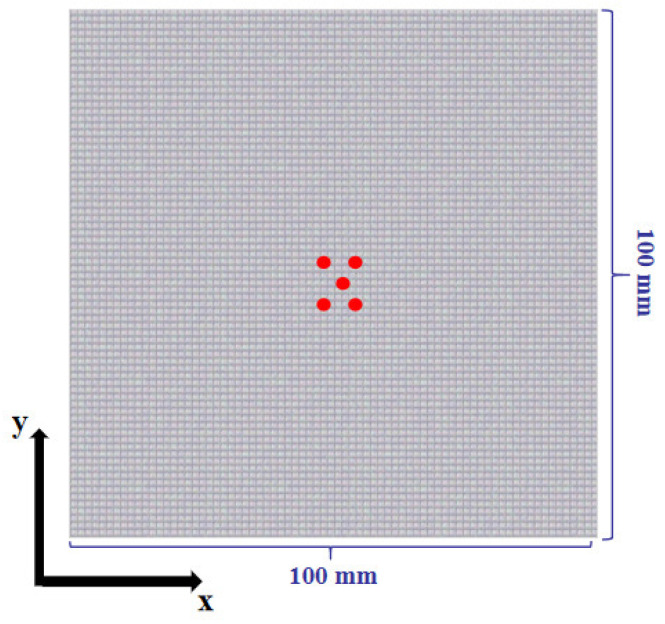
Temperature thermocouple arrangement of the cold surface.

During the test, two temperature thermocouples are placed in the center of the ceramic heater. One thermocouple is connected with a temperature controller to control the temperature of the heating plate, and the other thermocouple is used to record the temperature of the hot surface. Five temperature thermocouples are evenly placed in the center of the cold surface of the aerogel specimen (red circle), as shown in Figure 19. Finally, the average value of five temperature measuring points is selected as the cold surface temperature of the aerogels. The temperature of the ceramic heater is heated to 373 K, 573 K, and 873 K, respectively, by a temperature controller, and the test runs for 1100 s, thus recording the cold surface temperature of the aerogels.

## Figures and Tables

**Table 1:** Three testing methods of ETC.

Test Method		Disadvantage	Advantage
TCHM	SSHM	Long test time, unable to replicate real boundary conditions of thermal protection materials	High measurement accuracy
	THM	Large measurement error	Short test time
IRHM		Long test time, high cost and high result	High test temperature
CHM		Long test time, high cost	Realistic working boundary of thermal protection materials

**Table 2:** Material composition and basic characteristics of the silica aerogel composite.

Parameter	Description/Value
Material	Mullite-fiber-reinforced silica aerogel composite
Aerogel matrix	SiO_2_ aerogel
Reinforcement phase	Mullite fiber felt
Fiber type	Synthetic ceramic fiber
Main composition of fiber	Al_2_O_3_-SiO_2_
Continuous service temperature	~1473 K
Apparent density	373 kg/m^3^
Skeletal density	2466.4 kg/m^3^
Porosity	84.9%
BET surface area	568.7 m^2^/g
Average pore size	16 nm

**Table 3:** Thermophysical parameters of metal and aerogel.

	Parameter	Density (kg/m^3^)	Specific Heat Capacity (J/(kg·K))	Thermal Conductivity (W/(m·K))
Material				
Metel		8978	381	387.6
Aerogel		200	1000	0.02

**Table 4:** Specific information of test equipment.

Sensor	Accuracy	Model
Temperature controller	±0.1%	AI-719AX31L0S1
Power regulator	±0.5%	YS900Z-20040
Data acquisition instrument	±0.2%	Agilent 34972A
Thermocouple	±0.15 K	K type
$T_{\mathrm{avg}}$	0.9%	--

## Data Availability

The original contributions presented in this study are included in the article. Further inquiries can be directed to the corresponding authors.
